# Combination therapy with bevacizumab and a CCR2 inhibitor for human ovarian cancer: An in vivo validation study

**DOI:** 10.1002/cam4.5674

**Published:** 2023-02-22

**Authors:** Tianyue Zhai, Takashi Mitamura, Lei Wang, Shimpei I. Kubota, Masaaki Murakami, Shinya Tanaka, Hidemichi Watari

**Affiliations:** ^1^ Department of Obstetrics and Gynecology Hokkaido University Faculty of Medicine, Hokkaido University Hokkaido Sapporo Japan; ^2^ Institute for Chemical Reaction Design and Discovery (WPI‐ICReDD) Hokkaido University Hokkaido Sapporo Japan; ^3^ Department of Cancer Pathology Hokkaido University Faculty of Medicine, Hokkaido University Hokkaido Sapporo Japan; ^4^ Molecular Psychoimmunology, Institute for Genetic Medicine Graduate School of Medicine, Hokkaido University Hokkaido Sapporo Japan; ^5^ Group of Quantum immunology, Institute for Quantum Life Science National Institute for Quantum and Radiological Science and Technology (QST) Chiba Japan; ^6^ Division of Molecular Neuroimmunology, Department of Homeostatic Regulation, National Institute for Physiological Sciences National Institutes of Natural Sciences Okazaki Japan; ^7^ Institute for Vaccine Research and Development (HU‐IVReD) Hokkaido University Sapporo Japan

**Keywords:** angiogenesis, antiangiogenic drug, bevacizumab, CCR2 inhibitor, preclinical drug evaluation

## Abstract

**Background:**

Anti‐angiogenic therapy with bevacizumab (BEV), an anti‐VEGF antibody, plays a critical role in the treatment of ovarian cancer. However, despite an encouraging initial response, most tumors become resistant to BEV over time, and a new strategy that enables sustainable treatment using BEV is therefore needed.

**Methods:**

To overcome the resistance to BEV in patients with ovarian cancer, we performed a validation study of combination therapy with BEV (10 mg/kg) and the CCR2 inhibitor BMS CCR2 22 (20 mg/kg) (BEV/CCR2i) using 3 consecutive patient‐derived xenografts (PDXs) of immunodeficient mice.

**Results:**

BEV/CCR2i demonstrated a significant effect of growth suppression in the BEV‐resistant serous PDX and BEV‐sensitive serous PDX compared with BEV (30.4% after the second cycle and 15.5% after the first cycle, respectively), and treatment cessation did not attenuate this effect. Tissue clearing and immunohistochemistry with an anti‐α‐SMA antibody suggested that BEV/CCR2i suppressed angiogenesis from the host mice more than BEV. In addition, human CD31 immunohistochemistry revealed that BEV/CCR2i decreased microvessels originating from the patients to a significantly greater degree than BEV. Regarding the BEV‐resistant clear cell PDX, the effect of BEV/CCR2i was unclear during the first five cycles, but the following two cycles of increased‐dose BEV/CCR2i (CCR2i 40 mg/kg) significantly suppressed tumor growth compared with BEV (28.3%) by inhibiting the CCR2B‐MAPK pathway.

**Conclusions:**

BEV/CCR2i showed a sustained anticancer immunity‐independent effect in human ovarian cancer that was more significant in serous carcinoma than in clear cell carcinoma.

## Graphical abstract

Combination with bevacizumab and CCR2 inhibitor demonstrated a sustained synergistic effect. The effect mechanisms included inhibitions of both angiogenesis and the CCR2B‐MAPK pathway. The effect mechanisms were independent of antitumor immunity.

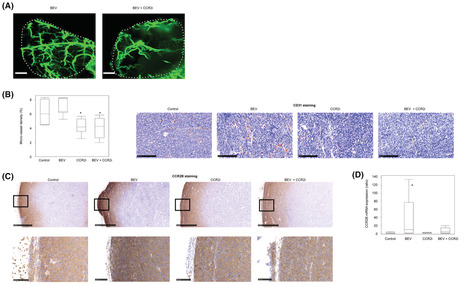

## 1 INTRODUCTION

Angiogenesis is a crucial stage in the development and spread of tumors. The development of antiangiogenic cancer medicines has been aided by the successful reduction in human tumor xenograft growth using a monoclonal antibody specific for vascular endothelial growth factor (VEGF).^1^ The US Food and Drug Administration (FDA) authorized bevacizumab (BEV) as the first anti‐VEGF medication for cancer patients in 2004. Patients with metastatic ovarian cancer,^2^, ^3^, ^4^ colorectal cancer,^5^ lung cancer,^6^ kidney cancer,^7^ and glioblastoma were successfully treated with BEV therapy in phase 3 clinical trials.^8^ Following the success of BEV, other antiangiogenic agents with different mechanisms have been developed.^9^

Over 200,000 women each year die from ovarian cancer, the most lethal malignancy of the female reproductive system.^10^ The standard treatment is primary debulking surgery to achieve no gross residual tumor followed by adjuvant platinum‐based chemotherapy.^11^ In addition, adjuvant platinum‐based chemotherapy with concurrent antiangiogenic agent BEV followed by single‐agent maintenance BEV is a recommended option for advanced ovarian cancer.^2^, ^3^ The survival advantages for patients receiving antiangiogenic therapy are minimal since, despite the initial response, the majority of tumors develop resistance to these medications. Therefore, a new strategy is needed to increase sensitivity to BEV by overcoming drug resistance.

C‐C motif chemokine receptor 2 (CCR2) is a receptor for C‐C motif chemokine ligand 2 (CCL2), a chemokine receptor that mediates monocyte chemotaxis and has two isoforms, CCR2A and CCR2B, with variations in its C‐terminal tails.^12^, ^13^ CCR2 is prominently expressed in lymphoid tissues, such as the tonsils, bone marrow, lymph nodes, and spleen. CCR2 is also expressed in non‐lymphoid organs including the uterine cervix, breast, and endometrium.^14^ Previous studies have shown that CCR2 is expressed in endothelial cells and involved in wound injury repair^15^ and vascular permeability.^16^ Furthermore, CCR2 is expressed in various human cancers, including skin, head and neck, glioma, and uterine cervical cancer.^17^ CCR2 and its ligands have been implicated in carcinogenesis,^18^ proliferation,^19^ metastasis,^20^ and angiogenesis.^21^, ^22^, ^23^, ^24^

Regarding angiogenesis, our field of our interest, a previous report showed that CCR2 was involved in tumor‐promoting angiogenesis and induction of resistance to antiangiogenic therapy.^25^ Based on this evidence, CCR2 has been expected to be a potential therapeutic target for human cancer. In two ongoing phase 2 clinical trials, the CCR2 inhibitor (CCR2i) BMS‐813160 was combined with the immune checkpoint inhibitor nivolumab and expected to show a significant immune response against non‐small‐cell lung cancer, hepatocellular carcinoma (NCT04123379), and renal cell carcinoma (NCT02996110). However, in previous clinical trials, ligand CCL2 inhibitors did not show promising efficacy against metastatic castrate‐resistant prostate cancer (NCT00992186). An animal experiment also showed that interruption of CCL2 inhibition by an anti‐CCL2 antibody paradoxically leads to an overshoot of metastases and accelerated death.^20^

These results led to several realizations. First, targeting receptor CCR2 for treatment is more promising than targeting ligand CCL2, which has several pitfalls in clinical settings. Second, we should wait for the results of ongoing clinical trials investigating significant anticancer immune responses to combination therapy with an immune checkpoint inhibitor and CCR2i. Third, overcoming resistance to antiangiogenic cancer therapy via CCR2 inhibition is expected.

The present study investigated the effect of combination therapy with a CCR2i and the anti‐VEGF antibody BEV. We used patient‐derived xenograft (PDX) models of human ovarian cancer for the evaluation, similar to the clinical setting.

## 2 MATERIALS AND METHODS

### 2.1 In vivo models

We asked the ovarian cancer patients treated at Hokkaido University Hospital to provide surgical specimens between 2019 and 2021. We included any patients as long as they were histologically diagnosed with epithelial ovarian cancer, regardless of treatment history, including antiangiogenic therapy, and set no particular exclusion criteria. Informed consent was obtained from all subjects.

To establish the PDX, we obtained the first and third high‐grade serous carcinoma specimens during surgery and immediately transplanted them into female NOD/Shi‐scid, IL‐2Rγ KO Jic mice (6‐week‐old and 18–22 g; CLEA Japan, Inc.,). We preserved the second clear cell carcinoma in liquid nitrogen and transplanted it another day. All tumors were cut into small pieces (<3 mm), with subcutaneous grafting, and left until all tumor diameters had increased to approximately 10 mm. All mice were randomly assigned to each treatment group. All tumor volumes were measured under general anesthesia using isoflurane inhalation (Wako) and calculated using the following formula: volume (mm^3^) = most extended diameter × shortest diameter × thickness. Mice were housed in a pathogen‐free environment and kept on a 12 h light–dark cycle at a temperature of 23 to 25°C with unrestricted access to food and double‐distilled water.

Peritumoral injection, intraperitoneal injection (IP), and intravenous injection (IV) are representative methods for drug administration in animal experiments. Because we set the primary purpose for investigating surface angiogenesis of xenografts with direct exposure to the experimental treatment and needed to randomize mice to match the tumor diameter for a fair evaluation before treatment without any visible markers, we chose subcutaneous transplantation and peritumoral drug injection. We referred to several previous reports for direct peritumoral injection.^26^, ^27^, ^28^

We administered the following treatments every 3 or 4 days by 2 diagonal injections of 25 μL of dissolved drugs directly to the tumor edge: CCR2i (low‐dose: 20 mg/kg or high‐dose: 40 mg/kg in DMSO, BMS CCR2 22; TOCRIS Bioscience, Bristol, UK), and BEV (10 mg/kg in saline; Chugai, Kanagawa, Japan). Cervical subluxation sacrifice therapies were used when the size of the tumor interfered with the mobility of the mice or when the condition of the mice deteriorated severely.

### 2.2 RNA isolation and quantitative reverse transcription polymerase chain reaction (qRT–PCR)

The frozen PDXs and cultured KF28 and RMG‐I cells, were used to extract total RNA. Following the manufacturer's instructions, total RNA was extracted using the RNeasy Mini Kit (Qiagen, Hilden, Germany) after the tissues and cells had been dissolved in TRIzol (Invitrogen). A NanoDrop 2000 spectrophotometer (Applied Biosystems; Thermo Fisher Scientific Inc.) was used to quantify RNA. The six TaqMan probes utilized in this study, as well as the 18 s rRNA used as endogenous control, were carefully chosen from the TaqMan gene expression assays (Applied Biosystems). 18 S (Assay ID: Hs99999901_s1), CCR2A (Assay ID: Hs00174150_m1), CCR2B (Assay ID: Hs00704702_s1), VEGFA (Assay ID: Hs00900055_m1), TGF‐β (Assay ID: Hs00998133_m1), and CCL2 (Assay ID: Hs00234140_m1).

The probe was employed in a 20 μL reaction with 10 μL TaqMan Fast Advanced Master Mix (Thermo Fisher Scientific Inc.,), 1 μL probe, 7 μL water, and 2 μL cDNA template. The StepOnePlus Real‐Time PCR system (Applied Biosystems) was used to analyze the data. All reactions were conducted using the following temperature profile: 50°C for 2 min (UNG incubation), 95°C for 20 s (polymerase activation), 40 cycles of 95°C for 1 s (denaturation), and 60°C for 20 s (annealing, extension). The 2^−ΔΔCt^ approach was used to relatively quantify the expression of the target gene.

### 2.3 Immunohistochemistry (IHC)

The xenografted tumors were removed, fixed with 10% formaldehyde, embedded in paraffin, and sectioned into 4‐μm‐thick sections. Slides were incubated with primary antibodies against CD31 (1:1000 dilution, Cat# 11265‐1‐AP, RRID:AB_2299349; Proteintech), CCR2A (1:200 dilution, Cat# 16153‐1‐AP, RRID:AB_2262945; Proteintech), and CCR2B (1:300 dilution; Cat# 16154‐1‐AP, RRID:AB_2878224; Proteintech). For microscopic observation, the sections were incubated with diaminobenzidine (DAB) chromogen and counterstained with hematoxylin. CD31 is commonly used to detect the presence of endothelial cells in histological sections and assess the extent of tumor angiogenesis.^29^ In the microvessel density (MVD) evaluation, we calculated the MVD of representative 10 regions for each tumor tissue section using a high‐power field of view (× 10) and used the mean values for the statistical analysis.

### 2.4 Western blotting

Tumor tissues were lysed in RIPA buffer (10 mM Tris [pH 7.4], 150 mM NaCl, 1% Triton X‐100, 1% sodium deoxycholate, 5 mM EDTA, 10% glycerol, 0.1% sodium dodecyl sulfate [SDS]) to prepare cell lysates for Western blot testing. We collected the supernatant following homogenization with a Polytron homogenizer and centrifugation at 15000 g for 10 min at 4°C. Using the Protein Assay Dye Reagent Concentrate (Bio‐Rad Laboratories, Hercules, CA, USA), the protein concentrations in whole‐cell lysates were evaluated, and equal protein quantities (20 μg) were then heated to 95°C for 10 min in SDS sample buffer (25 mL glycerol, 31.2 mL Tris buffer, 7.5 mL SDS, and a dash of bromophenol blue/100 mL). SDS‐polyacrylamide gel electrophoresis (PAGE), which separated proteins using a 15% SDS sulfate‐polyacrylamide gel, was used to transfer the proteins to PVDF membranes (Millipore, Billerica, MA, USA). Membranes were blocked in 5% non‐fat dry milk in TBS‐T buffer (0.1% Tween‐20 in Tris‐buffered saline) for 1 h at room temperature and then exposed to primary antibodies against CCR2A (1:1000 dilution, Cat# 16153‐1‐AP, RRID:AB_2262945; Proteintech), and CCR2B (1:1000 dilution; Cat# 16154‐1‐AP, RRID:AB_2878224; Proteintech), p44/42 MAPK (Erk1/2, 1:1000 dilution, Cat# 9102, RRID:AB_330744; Cell Signaling Technology), phosphor‐p44/42 MAPK (Erk1/2 Thr202/Tyr204, 1:1000 dilution, Cat# 9101, RRID:AB_331646; Cell Signaling Technology), and anti‐actin (1:1000 dilution, Cat# MAB1501, RRID:AB_2223041; Millipore) in 5% milk/TBS‐T or 5%BSA/TBS‐T overnight at 4 °C. The membranes were rinsed with TBS‐T buffer at least thrice for 10 min after shaking. Membranes were coated with a horseradish peroxide‐conjugated secondary antibody at a dilution of 1:5000 in 5% milk/TBS‐T for 1.5 h at room temperature. Following washing, the ECL reagent (Amersham, GE Healthcare) was used to create signals on the membrane, and the ChemiDoc system (Bio‐Rad) was used to photograph the results. The bands were seen using SuperSignal West Femto Maximum Sensitivity Substrate (Thermo Fisher Scientific, Inc.).

### 2.5 Cell culture

Dr. Yoshihiro Kikuchi kindly provided human KF28 (RRID:CVCL_V533) ovarian cancer cells. RMG‐I human ovarian clear cell carcinoma and HUVEC human endothelial cell lines were obtained from the JCRB Cell Bank (RRID: CVCL_1662) and the Riken Cell Bank (Tsukuba, Japan; RRID: CVCL_2959). KF28 cultures were grown in DMEM (Sigma‐Aldrich, St. Louis, MO, USA), RMG‐I cells in Ham's F12 media (Sigma‐Aldrich), and EBM‐2 medium (Lonza, Walkersville, MD, USA) was used to cultivate HUVECs. All media were prepared with 10% fetal bovine serum (Sigma, Aldrich), 2 mM L‐glutamine (Lonza), and 100 U/mL penicillin/streptomycin (Lonza) and were then incubated at 37°C in a 5% CO_2_ atmosphere.

### 2.6 Tissue clearing for PDXs

The fixed tumors were cleared by the CUBIC protocol^30^ and stained with monoclonal anti‐actin, α‐smooth muscle‐FITC antibody (F3777; Sigma‐Aldrich) as previously reported.^31^ Three‐dimensional images of tumors were acquired with a light‐sheet fluorescence microscope (UltraMicroscope II; Miltenyi Biotec, Germany) and analyzed with the Imaris software program (version 9.6.0; Bitplane AG).

### 2.7 MTT assay

The MTT assay for cytotoxicity was performed to the manufacturer guidelines using a Cell Proliferation Kit I (Roche, Mannheim, Germany). In summary, 7500 cancer cells were seeded per well in 96‐well plates in 100 μL of culture medium with reagents. We increased the concentration of CCR2i 10‐fold from 10 to 10,000 nM. As a control, an equivalent volume of DMSO was employed. The absorbance of the formazan product was measured at a 550 nm wavelength using a microplate reader.

### 2.8 Invasion assay

HUVECs were seeded on Corning Matrigel 24‐Well Plate 8.0 micron (BioCoat) and cultivated for 72 h at 37 °C in a 5% CO_2_ atmosphere. Invading HUVECs (20 × 10^3^ cells/well) were counted, and KF28 (50 × 10^3^ cells/well) or RMG‐I (50 × 10^3^ cells/well) cells were introduced into the lower chamber with 500 μL of 1% FBS medium with or without CCR2i (1 × 10^3^ nM) as a chemoattractant. A light microscope was used to count invasive cells that had migrated through the membrane to the bottom surface in three distinct fields at a magnification of 100. Each experiment was conducted in triplicate.

### 2.9 Statistical analyses

Statistical analyses were performed using the JMP Pro 16 statistical software package (SAS Institute). Continuous variables were compared using Student's t‐test if normally distributed. Differences in variables that were not normally distributed were compared using the nonparametric Mann–Whitney U test. The MVD and CCR2B *mRNA* expression are presented as box plots, and other data are presented as means ± standard deviations. All statistical tests were two‐sided, and *p* < 0.05 were considered significant.

## 3 RESULTS

### 3.1 Effects of a CCR2i in combination with BEV on growth of ovarian cancer PDXs

During the study period of 2.5 years, we obtained 8 total surgical specimens. We transplanted all specimens into mice, and the xenografts grew in 3 (37.5%) (Table S1). To determine whether combining CCR2is with BEV enhanced the antiangiogenic effect, we treated these three consecutive PDXs (Table 1 and Figure S1).

**TABLE 1:** Clinical characteristics of the three patients.

PDX	Histological subtype	Age	Sex	Chemotherapy before sampling	Sampling method	Stage
1	High‐grade serous carcinoma	70	Female	None	Exploratory laparotomy before neoadjuvant chemotherapy	IVB
2	Clear cell carcinoma	58	Female	Resistant to the combination therapy of paclitaxel, carboplatin, and bevacizumab	Metastatic skin tumor	IC
3	High‐grade serous carcinoma	61	Female	None	Exploratory laparotomy before neoadjuvant chemotherapy	IIIC

**FIGURE 1:**
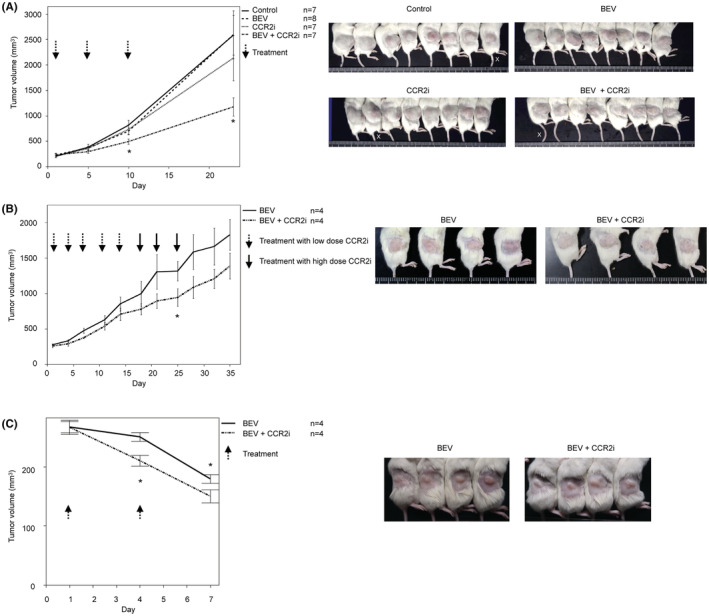
PDX treatment with CCR2 inhibitor and BEV. (A) Advanced high‐grade serous carcinoma. Left graph: Tumor volume change. Tumor volumes are shown as the mean ± standard error. *Significant difference. Right panels: Representative images of the tumors on Day 23. X: We excluded one mouse from the control, CCR2i, and BEV/CCR2i groups due to outlying data. (B) Metastatic clear cell carcinoma. Left graph: Tumor volume change. Tumor volumes are shown as mean ± standard error. *Significant difference. Right panels: Representative images of the tumors on day 35. (C) Advanced high‐grade serous carcinoma. Left graph: Tumor volume change. Tumor volumes are shown as mean ± standard error. *Significant difference. Right panels: Representative images of the tumors on day 7.

The first PDX was a high‐grade serous carcinoma of the primary tumor before chemotherapy, and whether or not this patient was resistant to BEV was unknown. Eight mice were treated with BEV, a low‐dose CCR2i, a combination of BEV and a low‐dose CCR2i (BEV/low‐dose CCR2i), or solutions alone (Control) from day 1 (Figure 1A). One mouse each from the control, low‐dose CCR2i, and BEV/low‐dose CCR2i groups were excluded from the analysis due to outlying data for the tumor volume.

After the second treatment (Day 10), the tumor volumes in the control, BEV, and low‐dose CCR2i groups increased and were not significantly different among the three groups, demonstrating that single‐agent BEV or low‐dose CCR2i did not have a therapeutic effect on PDX. By contrast, BEV/low‐dose CCR2i significantly suppressed tumor growth compared with BEV (30.4% reduction*, p* = 0.03). To investigate whether or not low‐dose BEV/CCR2i had a long‐term effect, we administered another treatment on Day 10 and observed the tumor volume. Interestingly, PDX growth suppression in the BEV/low‐dose CCR2i group was maintained for 2 weeks (on day 23) compared with that in the control group (54.5% reduction, *p* = 0.01), while the BEV group did not show any effect (0.0% reduction, *P* = 0.99) and the low‐dose CCR2i group also showed a limited effect (17.3% reduction, *P* = 0.46). These results suggest that the treatment effect of BEV/low‐dose CCR2i on primary BEV‐resistant ovarian cancer was synergistic, and importantly, this effect was sustained after treatment cessation.

Next, we established a second PDX for clear cell carcinoma to confirm the effect of BEV/CCR2i in another BEV‐resistant patient. Because the first PDX model showed that a single agent CCR2i did not show tumor growth suppression in some patients, we omitted the CCR2i group. In addition, we left the BEV group out because we knew that the second patient was clinically BEV‐resistant. We then narrowed the treatment groups from four with the first PDX to two: BEV (*n* = 4) and low‐dose BEV/CCR2i (*n* = 4) (Figure 1B). Although the mean tumor volume in the BEV/low‐dose CCR2i group was always lower than that in the BEV group after five treatment cycles, we found no significant difference in tumor volume between the two groups until Day 18. To investigate whether or not dose escalation of CCR2i showed an increased therapeutic effect, we added two additional cycles of high‐dose CCR2i. On Day 25, the tumor volume was significantly lower in the BEV/high‐dose CCR2i group than in the BEV group (28.3% reduction, *p* = 0.046). The tumor volume remained lower in the BEV/high‐dose CCR2i group than in the BEV group after the third additional cycle (Day 25) until Day 35. These results suggest that some patients are resistant to BEV/CCR2i, possibly due to differences in histological subtypes.

We then established a third PDX for high‐grade serous carcinoma using a primary tumor before chemotherapy. We treated the PDX with BEV and BEV/low‐dose CCR2i (Figure 1C), similar to the second PDX. This PDX was sensitive to BEV, and the tumor volume decreased immediately after the first cycle. Interestingly, BEV/CCR2i showed a powerful additive effect after the first and second cycles (28.3% and 19.3% reduction compared with BEV, *p* = 0.01 and 0.03, respectively, on days 3 and 7).

During treatment with these three PDXs, we observed no severe adverse effects associated with bleeding, intestinal perforation, or treatment‐related death.

### 3.2 Mechanisms underlying BEV/CCR2i treatment

Although BEV/CCR2i was effective in human ovarian cancer, the following questions remained: (1) “Is the mechanism of BEV/CCR2i limited to angiogenesis in the host mice?” and (2) “Does the difference in the sensitivity of BEV/CCR2i depend on histology?” Basic experiments were thus conducted to address these questions.

**FIGURE 2:**
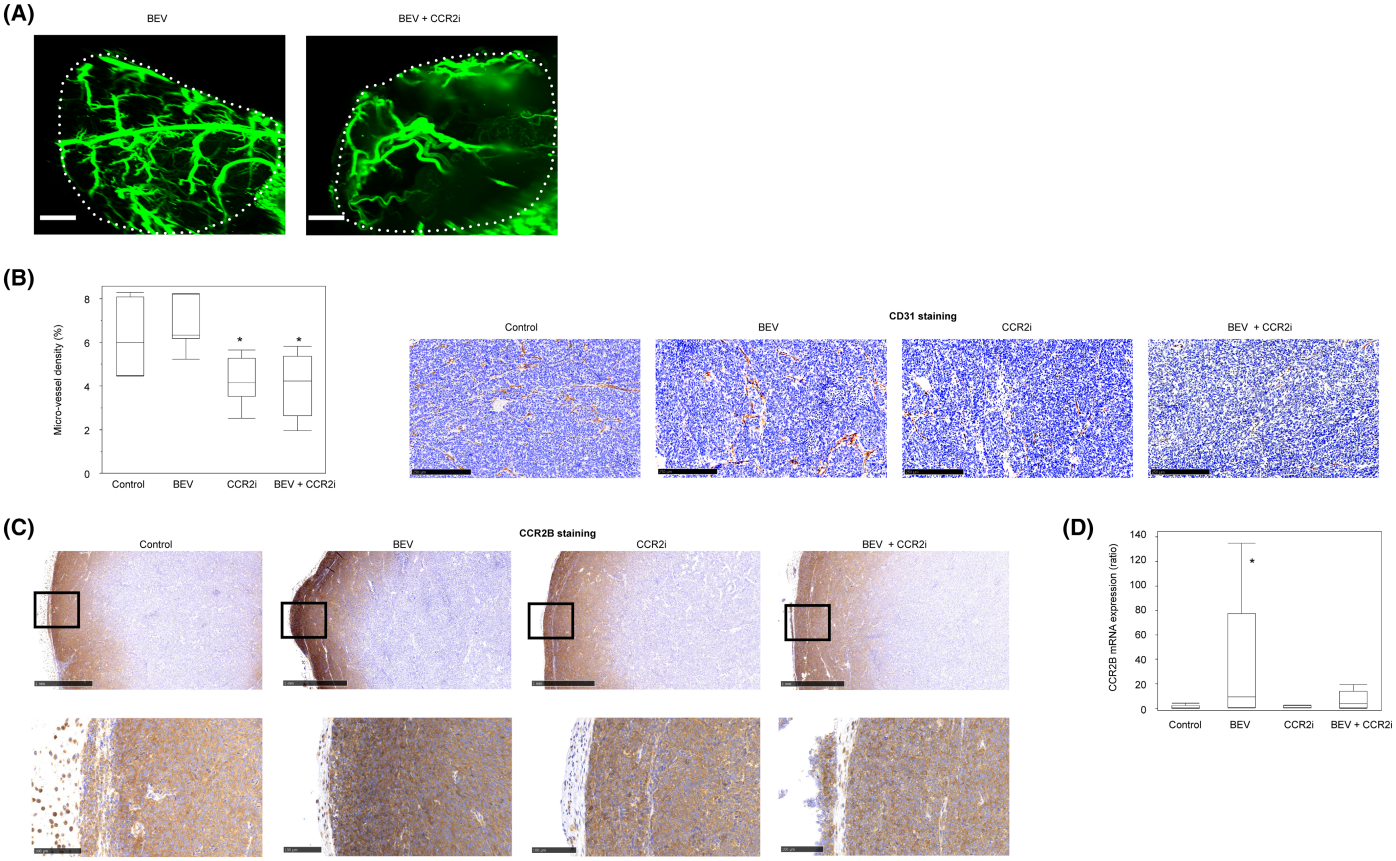
Combination therapy with BEV and a high‐dose CCR2i suppressed tumor growth by inhibiting the CCR2B‐MAPK pathway. (A) Representative image of the mouse blood vessel structure on the tumor surface in each treatment for the third PDX. α‐smooth muscle actin is stained in green fluorescence. The white bar shows a length of 1 mm, and the broken white lines indicate the tumor edges. (B) Left box plot: density of xenograft‐derived microvessel in each treatment for the third PDX, *: a significant difference. Right images: representative human CD31 staining in each treatment for the first PDX. The black bar shows a length of 250 μm. (C) CCR2B expression. Representative images of CCR2B staining in each treatment for the first PDX. Left top: low‐power field (× 40). The black bar shows a length of 1 mm. Left bottom: high‐power field (× 100). The black bar shows a length of 100 μm. Right box plot: RT–PCR analysis of *CCR2B* mRNA expression. *: a significant difference.

We first examined the CD31, CCR2A, and CCR2B expression in the blood vessels of the first serous PDX and found that all three were expressed in endothelial cells (Figure S2). To confirm that BEV/CCR2i suppressed angiogenesis, we made the blood vessels of the third serous PDX visible by tissue clearing and IHC using an anti‐α‐SMA antibody. The number of blood vessels on the tumor surface, the front of angiogenesis, was decreased in the BEV/CCR2i group compared with the BEV group (Figure 2A). Although we did not quantify the blood vessel density of the whole tumor, this result suggests that the critical mechanism underlying BEV/CCR2i treatment is its antiangiogenic effect in host mice. We then investigated whether or not the microvessels originating from the patients had changed. The human CD31‐positive MVD was not markedly different between the control and BEV (Figure 2B), whereas the MVD was significantly decreased in CCR2i and BEV/CCR2i compared with BEV (*p* = 0.002 and *p* = 0.004, respectively). This result shows that CCR2i used alone or in combination with BEV decreased the number of human blood vessels in xenografts. These findings suggested that BEV/CCR2i inhibited angiogenesis in host mice and maintained human blood vessels in xenografts.

To confirm whether or not the CCR2i had a direct suppressive effect on CCR2‐positive cancer cells other than its antiangiogenic effect, we examined the CCR2 expression in the first serous PDX using IHC. The CCR2A expression did not differ markedly among the control, BEV, CCR2i, and BEV/CCR2i groups (Figure S3). By contrast, CCR2B was expressed in cancer cells at the tumor edges, and staining was most robust in the BEV group and suppressed in the BEV/CCR2i group (Figure 2C). The *CCR2B* mRNA expression, quantified by real‐time PCR using whole tumors, was increased in the BEV group by 40‐fold compared to that in the control group (*P* = 0.03) and, as expected from IHC, decreased in the BEV/CCR2i group to the level of the control (no statistical difference). These results suggest that BEV strongly induces CCR2B in ovarian cancer cells and that CCR2i has a direct suppressive effect on cancer cell viability via CCR2B.

**FIGURE 3:**
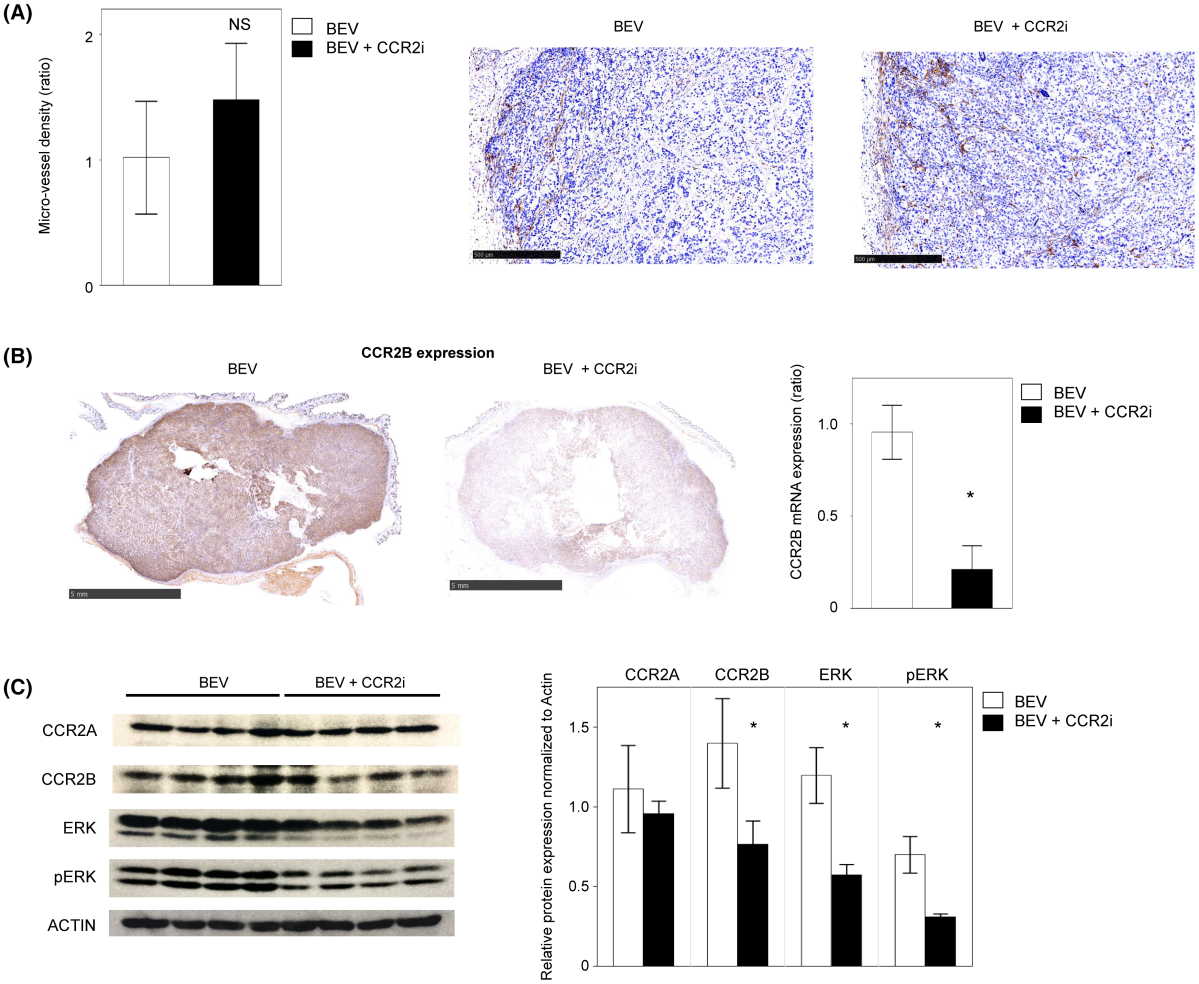
CCR2i suppressed the growth of tumors resistant to combination therapy with BEV and the CCR2i by direct inhibition of the CCR2‐MAPK pathway. (A) Left bar graph: density of xenograft‐derived microvessels in each treatment for the second PDX. Data are shown as the means ± standard error of triplicate assays. Right pictures: representative images of CD31 staining in each treatment for the second PDX. (B) Left images: representative pictures of CCR2B staining in each treatment for the second PDX. The black bar shows a length of 5 mm. Right graph: Results of an RT–PCR analysis of the *CCR2B mRNA* expression. Data are shown as the means ± the standard error and *: a significant difference. (C) Left images: immunoblot of CCR2A, CCR2B, ERK, phosphorylated ERK (pERK), and actin expression in each treatment (left four: BEV, right four: BEV + CCR2i) for eight samples from the second PDX. Right graph: normalized CCR2A, CCR2B, ERK, and phosphorylated ERK (pERK) expression in each treatment for the second PDX. Data are shown as the means ± the standard error. *: a significant difference.

To determine whether or not the difference in sensitivity to BEV/CCR2i was due to the histological subtype, we examined the blood vessels of the second PDX of clear cell carcinoma. The number of peritumoral blood vessels was low in this model (data not shown). Interestingly, there was no marked difference in the density of human vessels in the tumors between BEV and BEV/CCR2i cells (Figure 3A). However, the total volume of the tumors did decrease. Therefore, we hypothesized that the CCR2i had some type of biological activity that directly inhibited the tumor cell activity in addition to its antiangiogenic effect. IHC showed the CCR2A expression did not differ markedly between the BEV and BEV/CCR2i groups (Figure S4). By contrast, CCR2B was expressed in cancer cells of the whole tumor and was notably suppressed in BEV/CCR2i compared with BEV (Figure 3B, left). The *CCR2B mRNA* expression was then quantified using real‐time PCR of tumor tissue specimens, and the BEV/CCR2i expression was thus found to have significantly decreased (*p* = 0.03, Figure 3B, right). Western blotting of whole tumors showed that BEV/CCR2i selectively suppressed the CCR2B isoform (Figure 3C, *p* = 0.03) compared with BEV. Downstream of CCR2B, phospho‐ERK was significantly decreased in BEV/CCR2i compared with BEV (*p* = 0.03). Furthermore, total ERK was also decreased in BEV/CCR2i compared with BEV (*P* = 0.03).

These results suggest that CCR2is have diverse effects on ovarian cancer, including sensitization to BEV antiangiogenic therapy and direct suppression of the CCR2B pathway in cancer cells.

### 3.3 Suppressive effect of CCR2is on endothelial and cancer cell viability in vitro

**FIGURE 4:**
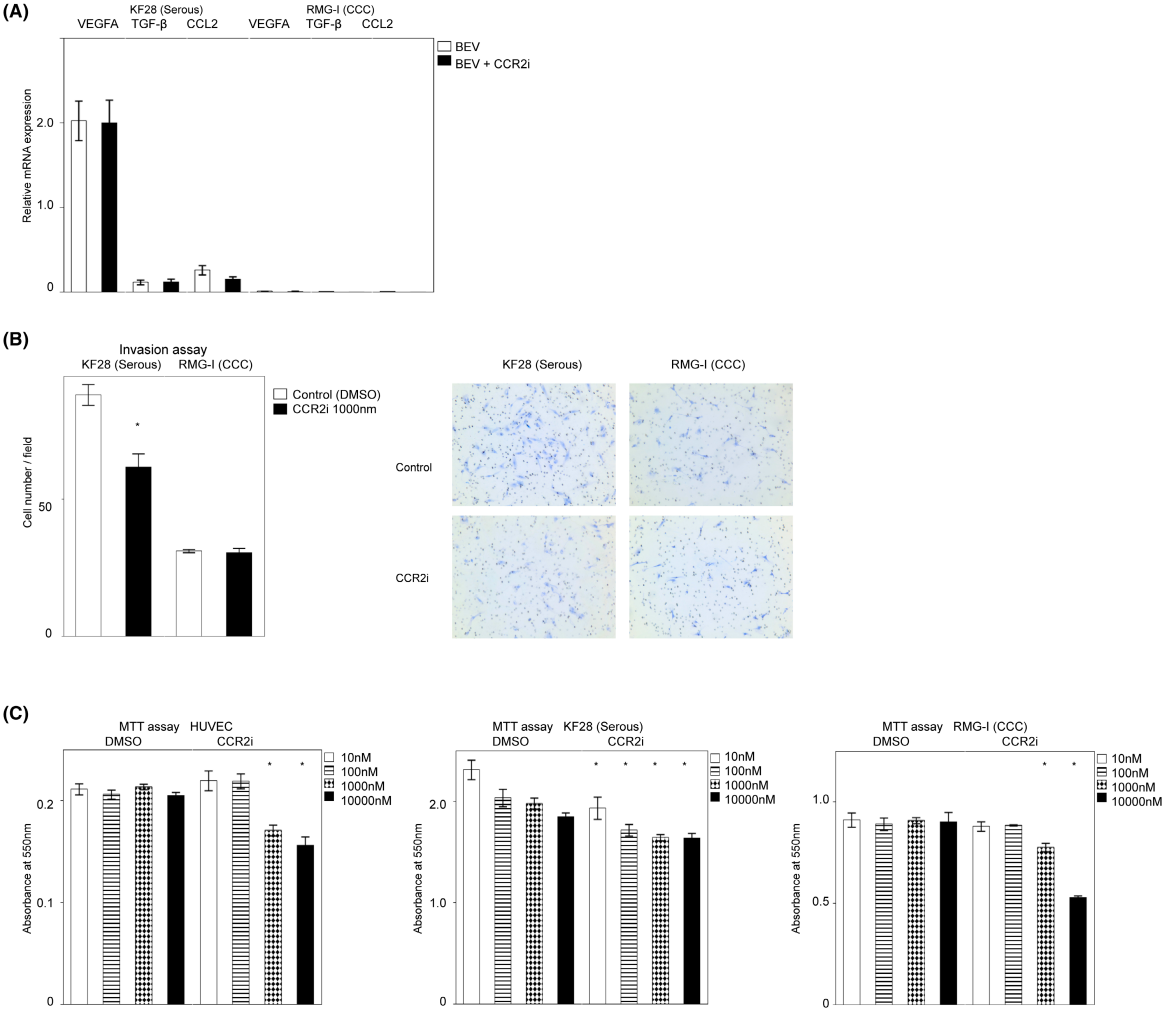
Histological subtype of ovarian cancer is involved in the CCR2i response. (A) Results of an RT–PCR analysis of pro‐angiogenic factors' expression under BEV ± CCR2i treatment. Data are shown as the means ± the standard error of triplicate assays. (B) Invasion assay. Left bar graph: cell numbers of KF28 or RMG‐I‐induced invasion of HUVECs in each assay in representative high‐power fields. Data are shown as the means ± the standard error of triplicate assays. Right pictures: representative images of invasion in each assay in high‐power fields (×100). *: a significant difference. (C) MTT assay. Bar graphs show absorbance at 550 nm. Data are shown as the means ± the standard error of triplicate assays. *: a significant difference.

To elucidate whether the antiangiogenic effect of CCR2i was indirect via cancer cells or direct via endothelial cells, we performed several in vitro assays. The *mRNA*s of the pro‐angiogenic factors *VEGFA*, *TGF‐*β, and *CCL*2 were exclusively expressed in the KF28 serous carcinoma cell line compared with the RMG‐I clear cell carcinoma cell line (Figure 4A) suggesting that the angiogenesis dependency of the first and third serous PDX, which were sensitive to a lower concentration of CCR2i, was higher than that of the second clear cell PDX. In addition, the invasion of endothelial cells co‐cultured with KF28 was significantly attenuated when KF28 was treated with a CCR2i (*p* < 0.01), whereas there was no marked difference in endothelial cells co‐cultured with RMG‐I regardless of CCR2i treatment (Figure 4B). These results suggest that CCR2is exert an indirect antiangiogenic effect by suppressing the secretion of angiogenic factors from cancer cells.

Next, we determined whether or not the viability of endothelial and cancer cells was directly attenuated by CCR2is (Figure 4C) in vitro. The MTT assay showed that serous KF28 was the most sensitive, and low‐dose CCR2i (10 nM) significantly decreased the cell viability (*p =* 0.01), whereas the viability of clear cell RMG‐I was decreased by high‐dose CCR2i (*p* < 0.01, over 1000 nM), which was similar to of the findings with HUVECs (*p* < 0.01, than 1000 nM). These results suggest that CCR2is directly decrease cancer cell and endothelial cell viability, with the sensitivity depending on the cell subtype, so dose escalation of CCR2is may be effective in some CCR2i‐resistant patients.

## 4 DISCUSSION

This study demonstrated for the first time the synergistic or additive effect of BEV and a CCR2i in ovarian cancer patients using a PDX immune‐deficient mouse model. Adding a CCR2i prevented resistance to BEV, and importantly, tumor growth suppression was maintained for a long time after the cessation of treatment.

A previous clinical trial showed that the survival outcome was improved by BEV, but ultimately deteriorated to the same level as a placebo and even worsened after the cessation of treatment in some patients.^3^ In addition, a recent clinical trial showed that extended treatment with BEV (15–30 months) did not improve the survival of ovarian cancer patients.^32^ In a recent clinical study, considered the combination of BEV with another anticancer drug for BEV‐resistant ovarian cancer. The found that the median progression‐free survival (PFS) assessed in each group was 3.1 and 4.0 months (*p* = 0.0082).^33^ Although meaningful results were obtained, the improvement in the PFS from the patient's perspective was minimal. Therefore, in contrast to other studies, our study considered the development of new treatments rather than the readiministration of BEV. The combination of a CCR2i with BEV will be a new strategy that enables sustainable effects.

We also showed that this combination therapy had a powerful effect on a BEV‐sensitive patient (third PDX), suggesting that this strategy may be helpful for all‐comer ovarian cancer patients, regardless of treatment history or biomarkers.

We used a PDX model with genetically modified immunodeficient mice to enable xenograft transplantation. CCR2 plays an essential role in the immune system, particularly in monocytes. Previous studies have shown that the CCL2/CCR2 axis is a critical chemokine signaling pathway that creates the tumor microenvironment through the recruitment of immunosuppressive cells.^34^ As mentioned in the introduction, several ongoing clinical trials involving the combination of a CCR2i and immune checkpoint inhibitors are expected to show an advantage in anti‐cancer immunity as well as promising results in so‐called immunologically hot tumors, in which immune cells actively fight cancer cells. By contrast, our study suggests that such combination therapy is effective in immunologically cold tumors and complements the therapeutic role of the CCR2i.

In the second PDX experiment, we used a high dose of CCR2i to treat resistant tumors. However, this method may not be suitable in clinical settings in terms of adverse effects. Previous reports have described several methods for optimizing CCR2i delivery. For example, polyethylene glycol (PEG) nanoparticles of a CCR2 antagonist may help maintain the half‐life of the drug.^35^ VCAM‐1 targeted delivery of liposomes loaded with a CCR2 antagonist enabled specific delivery to the cancer cell‐activated endothelium^36^ In addition, lipid micelles composed of PEG‐distearoylphosphatidylethanolamine have high biocompatibility and can solubilize hydrophobic CCR2 inhibitors.^37^ These technologies will help to establish an efficient CCR2 inhibitor protocol with relatively few adverse effects in clinical practice.

Previous studies have shown that human endothelial cells express CCR2,^38^ and our findings regarding the antiangiogenic effects of a CCR2i on PDXs make sense. By contrast, our in vivo and in vitro experiments suggested that the CCR2i directly attenuated cancer cell viability. Notably, the CCR2B isoform is critical, as its expression exclusively decreases when CCR2 inhibition reduces phosphorylation of downstream MAPK. The functional differences between CCR2 isoforms in human cancer cells and details of the change in downstream signal pathways by CCR2i remain unclear. In particular, further studies involving comprehensive gene expression analyses are needed to confirm whether or not tumors in which the expression of both total ERK and pERK are decreased after BEV/CCR2i treatment start to depend on other pathways.

Chemotherapy for patients with malignancies has limited efficacy, and predicting efficacy prior to treatment is difficult. Recently, cancer genomic analyses have been used to identify effective treatments, but the likelihood of finding an effective treatment remains low, at <10%.^39^ Therefore, if drug sensitivity testing can be performed using patient tumor specimens, as in this study, it may be possible to predict efficacy prior to treatment initiation. Nonetheless, this study has several issues, such as the inconsistent success rate of PDX production (25%–83%),^40^ and the need to sacrifice small animals. However, despite the small number of PDXs in our study, we used three consecutive xenografts, which made the results reliable. Additional studies using intravenous or oral routes are needed to consider clinical applications. In conclusion, our study suggested the inhibitory effect of combination therapy with BEV and CCR2i on both tumor angiogenesis and tumor cell proliferation.

## AUTHOR CONTRIBUTIONS

**Tianyue Zhai:** Conceptualization (equal); formal analysis (equal); investigation (equal); methodology (equal); writing – original draft (equal); writing – review and editing (equal). **Takashi Mitamura:** Conceptualization (equal); formal analysis (equal); investigation (equal); methodology (equal); writing – original draft (equal); writing – review and editing (equal). **Lei Wang:** Conceptualization (equal); investigation (equal); methodology (equal); writing – review and editing (equal). **Shimpei I Kubota:** Formal analysis (equal); investigation (equal); methodology (equal). **Msaaki Murakami:** Supervision (equal). **Shinya Tanaka:** Supervision (equal). **Hidemich Watari:** Supervision (equal).

## FUNDING INFORMATION

This work was supported by Japan Society for the Promotion of Science (JSPS) KAKENHI (Japan, Grant Number JP19K09818), Kobayasi Foundation (Japan), Women‘s health Integrative Network of Doctors (Japan), and Society for Women’s Health Science Research (Rohto Pharmaceutical Co., Ltd., Japan).

## CONFLICT OF INTEREST STATEMENT

The authors have no conflict of interest.

## ETHICS STATEMENT

Approval of the research protocol by an Institutional Reviewer Board: Hokkaido University. Informed Consent: The human‐derived tumor tissues used in this study were obtained with the written consent of the patients in accordance with the ethical guidelines of the World Medical Association (Declaration of Helsinki). Recognized in 2018. Registry and the Registration No. of the study: Registration ID: 18–0128. Animal Studies: This study was conducted in accordance with the principles of the Declaration of Helsinki and the Animal Care Guidelines for the Care and Use of Laboratory Animals at Hokkaido University Graduate School of Medicine.

## Supporting information


Figure S1–S4.

Table S1.

## Data Availability

The data generated in this study are available upon request from the corresponding author.
