# Supplementary figures and images for: Combination therapy with bevacizumab and a CCR2 inhibitor for human ovarian cancer: An in vivo validation study
Source: Cancer Med. 2023 Feb 22;12(8):9697–708. doi: 10.1002/cam4.5674 (PMC10166889; doi:10.1002/cam4.5674)

S1

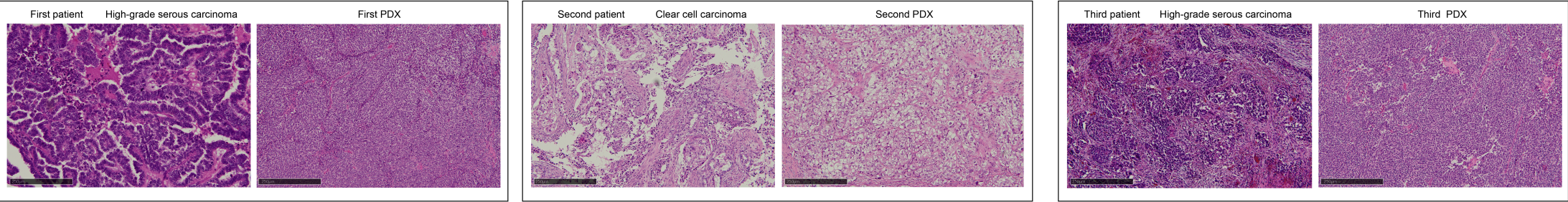

S2

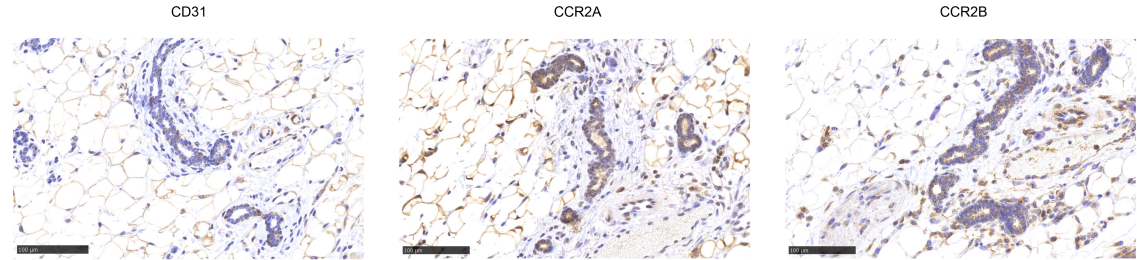

S3

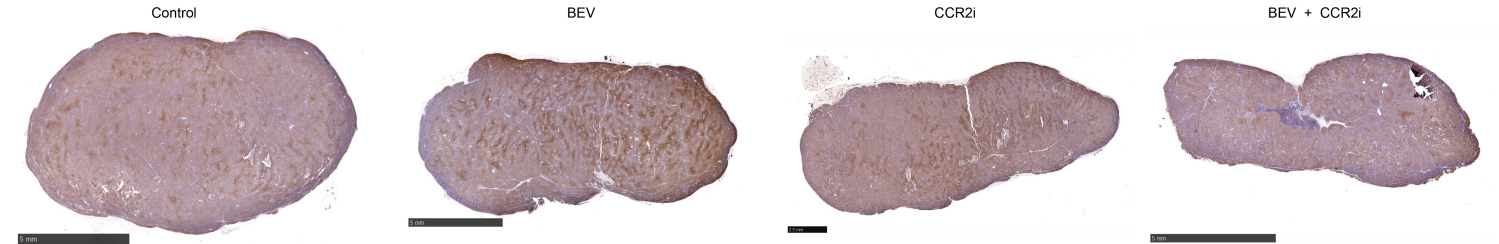

S4

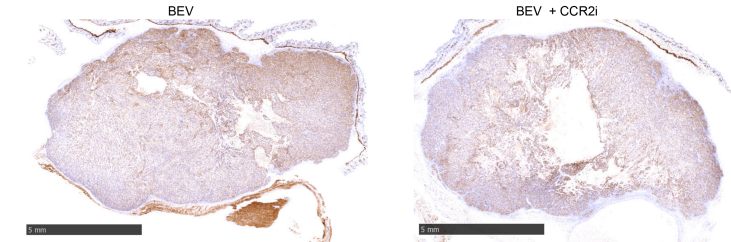

Supplement: Supplementary file 1 — Figure S1–S4. [file CAM4-12-9697-s002.pdf]
