# Supplementary material for: Combination therapy with bevacizumab and a CCR2 inhibitor for human ovarian cancer: An in vivo validation study
Source: Cancer Med. 2023 Feb 22;12(8):9697–708. doi: 10.1002/cam4.5674 (PMC10166889; doi:10.1002/cam4.5674)
Supplement: Supplementary file 2 — Table S1. [file CAM4-12-9697-s001.docx]

| **Supplementary Table.** Clinical characteristics of the five patients without successful PDX | | | | | |  |
| --- | --- | --- | --- | --- | --- | --- |
| PDX | Histological  subtype | Age | Sex | Chemotherapy before sampling | Sampling method | Stage |
| 1 | High-grade serous carcinoma | 61 | Female | None | Exploratory laparotomy before neoadjuvant chemotherapy | IIIC |
| 2 | High-grade serous carcinoma | 56 | Female | Sensitive to the combination therapy of paclitaxel, and carboplatin | Interval debulking surgery after neoadjuvant chemotherapy | IIIC |
| 3 | Clear cell carcinoma | 64 | Female | None | Primary debulking surgery | IC |
| 4 | High-grade serous carcinoma | 72 | Female | Sensitive to the combination therapy of paclitaxel, carboplatin, and bevacizumab | Interval debulking surgery after neoadjuvant chemotherapy | IIIC |
| 5 | Clear cell carcinoma | 47 | Female | None | Primary debulking surgery | IC |
